# Sendotypes predict worsening renal function in chronic kidney disease patients

**DOI:** 10.1002/ctm2.70279

**Published:** 2025-03-27

**Authors:** Thomas McLarnon, Steven Watterson, Sean McCallion, Eamonn Cooper, Andrew R. English, Ying Kuan, David S. Gibson, Elaine K. Murray, Frank McCarroll, Shu‐Dong Zhang, Anthony J. Bjourson, Taranjit Singh Rai

**Affiliations:** ^1^ School of Medicine Personalised Medicine Centre Ulster University Londonderry UK; ^2^ School of Health and Life Sciences Teesside University, Campus Heart Middlesbrough UK; ^3^ Western Health and Social Care Trust, Altnagelvin Area Hospital Londonderry UK

**Keywords:** biomarker, CKD, machine learning, sendotype, senescence

## Abstract

**Background:**

Senescence associated secretory phenotype (SASP) contributes to age‐related pathology, however the role of SASP in Chronic Kidney Disease (CKD) is unclear. Here, we employ a variety of omic techniques to show that senescence signatures can separate CKD patients into distinct senescence endotypes (Sendotype).

**Methods:**

Using specific numbers of senescent proteins, we clustered CKD patients into two distinct sendotypes based on proteomic expression. These clusters were evaluated with three independent criteria assessing inter and intra cluster distances. Differential expression analysis was then performed to investigate differing proteomic expression between sendotypes.

**Results:**

These clusters accurately stratified CKD patients, with patients in each sendotype having different clinical profiles. Higher expression of these proteins correlated with worsened disease symptomologies. Biological signalling pathways such as TNF, Janus kinase‐signal transducers and activators of transcription (JAK‐STAT) and NFKB were differentially enriched between patient sendotypes, suggesting potential mechanisms driving the endotype of CKD.

**Conclusion:**

Our work reveals that, combining clinical features with SASP signatures from CKD patients may help predict whether a patient will have worsening or stable renal trajectory. This has implications for the CKD clinical care pathway and will help clinicians stratify CKD patients accurately.

**Key points:**

- Senescent proteins are upregulated in severe patients compared to mild patients
- Senescent proteins can stratify patients based on disease severity
- High expression of senescent proteins correlates with worsening renal trajectories

## Graphical abstract

Graphical abstract of the CKD sendotype discovery pipeline demonstrating the use of multiple omic datasets from patient plasma, patient biopsies and kidney organoid models for both clustering and validation.

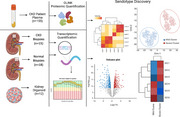

## 1 INTRODUCTION

Chronic kidney disease (CKD) is a debilitating condition that effects approximately 10% of the global population, with incidence rates increasing by upwards of 33% from 1990 to 2017.^1^, ^2^ It is defined clinically as having a glomerular filtration rate (GFR) less than 60 mL/min or an increased creatinine:albumin ratio above 30–300 mg/g:3–30 mg/mmol.^3^, ^4^ CKD can be staged according to the Kidney Disease Improving Global Outcomes (KDIGO) classification based on estimated GFR (eGFR), with stage 1 (≥90 mL/min/1.73 m^2^) and evidence of kidney damage, stage 2 (60–89 mL/min/1.73 m^2^) with evidence of kidney damage, stage 3a (45–59 mL/min/1.73 m^2^), stage 3b (30–44 mL/min/1.73 m^2^), stage 4 (15–29 mL/min/1.73 m^2^) and stage 5 (<15 mL/min/1.73 m^2^ or end‐stage renal disease [ESRD]).

CKD can be caused by various external stressors such as diabetes, hypertension, obesity, smoking, genetics factors and ageing.^4^, ^5^, ^6^ Additionally, renal ageing has been shown to be a staple mechanism in CKD pathophysiology as noted by a major reduction in GFR, creatinine clearance, proliferation of renal epithelial cells and number of functioning nephrons.^6^, ^7^, ^8^ There are many contributors of renal ageing that include an increase in antioxidants, fibrosis, telomere shortening and increased Wnt signalling^9^; however, an increase in cellular senescence is considered the main driver of renal ageing in the context of CKD pathophysiology.^10^, ^11^, ^12^

Senescence is the state of a cell when it no longer actively replicates. Senescence induction occurs through various stresses such as DNA damage, telomere erosion or when the Hayflick limit, the set number of times a primary cell is capable of division, is reached.^13^ Cells entering senescence can exhibit the senescence‐associated secretory phenotype (SASP), which causes senescent cells to secrete inflammatory markers. SASP can influence many chronic diseases, including cardiovascular disease (CVD),^14^ asthma,^15^, ^16^ coronavirus disease 2019^17^ and CKD.^18^, ^19^ Senescent cells also evade apoptosis through differential regulation of apoptotic factors relating to the senescent cell anti‐apoptotic pathways.^20^ Senescent cells can also chemo‐attract specific immune cells which direct their clearance; however, in dysfunctional tissue, senescent cells can accumulate and evade both innate and adaptive immune responses,^21^, ^22^ allowing them to continue promoting inflammation via SASP.

Increased levels of reactive oxygen species (ROS) as known signatures of senescence, contribute towards accelerated renal ageing,^23^, ^24^, ^25^ and are known to be further increased in CKD patients via indoxyl sulphate signalling cascades. Patients with CKD are known to have higher levels of indoxyl sulphate, a nephrotoxic metabolite that contributes towards chronic renal failure.^26^, ^27^ CKD patients cannot effectively filter out indoxyl sulphate, which binds to OAT1 and OAT3 receptors in turn activating the NADPH oxidase complex, resulting in increased levels of ROS.^24^, ^25^, ^26^, ^27^ These high levels of ROS initiate an inflammatory cascade which involves IkB kinase (IKK), Mitogen activated protein kinases (MAPK) Extra and nuclear factor‐kappa B (NF‐κB) signalling complexes, resulting in the release of many inflammatory cytokines and tumour necrosis factors, which is damaging for CKD patients.^28^

This creates a growing cycle of inflammation within CKD patients as ROS initiates inflammation and senescence causing renal injury through SASP,^8^, ^10^, ^11^, ^29^ which reduces renal filtration. Indoxyl sulphate levels increase, which causes further promotion of ROS. With senescence contributing towards functional decline and increased inflammation, it is possible that CKD has unique pathophysiological interactions with senescence in the form of disease subtypes. A disease subtype or endotype is a disease state with a different mechanism to the standard disease.^30^ Endotypes were first theorised in 2008 when studying asthma, as patient data suggested a lack of information that correlated phenotypes and symptomologies.^30^, ^31^ Since then, numerous chronic disease studies have employed the use of endotypes to better stratify patients and further understand disease pathophysiology and differing clinical symptomologies.^32^, ^33^, ^34^, ^35^ However, there is a lack of information regarding distinct endotypes of CKD and with patients displaying differing severities, symptomologies and trajectories.

We hypothesised that increased cellular senescence as a key factor in CKD is driving patients towards a worsened disease state, and since senescence plays such an important role in CKD, patients could be stratified into senescent endotypes or sendotypes,^36^ according to disease severity. The identification of potential sendotypes will further enhance our knowledge of disease progression in specific patients, discover potential drug targets to identify potential treatments and better identify patients more at risk of renal failure.

Here, we show that severe and mild CKD patients can be accurately separated using a small subset of plasma proteomics, with many proteins used being associated with senescence, resulting in the identification of a senescence signature that corresponds with worsening renal function, which we denote as a sendotype. We also show this sendotype correlates with worsening renal trajectories at 1‐year follow‐up recordings of creatinine and eGFR, suggesting high expression of senescent proteins may be responsible for differing biological events that contribute towards this observable sendotype. Lastly, we verified that the same enriched signalling pathways that these senescent signatures participate in are also enriched in kidney‐specific tissue through the analysis of independent biopsy and organoid transcriptomic datasets.

## 2 METHODOLOGY

### 2.1 Participant recruitment

One‐hundred and fifty‐five CKD patients (mean age = 59 years; 63% male) were recruited from Altnagelvin and Letterkenny University hospitals (approved by Northwest Research Ethics Committee; research protocol version 7.0 [01/08/2019]). CKD participants were recruited at outpatient clinics with disease stage ranging from 2 to 5 as per American Society of Nephrology. Of these 155 CKD participants, 79 participants were selected based on data availability, allowing the investigation to have the most detailed records with 1‐year follow‐ups of clinical recordings. Baseline recordings were conducted between 2018 and 2019, with 1‐year follow‐ups occurring between 2019 and 2020 (IRAS Project ID: 243910).

### 2.2 Proteomic quantification using proximity extension assay (OLINK proteomics)

Five‐hundred and thirty‐five proteins were measured using 5 µL of plasma per participant using the Proseek Multiplex proximity extension assay (PEA) (Olink Bioscience) on the Olink Multiplex Plates: cardiovascular panel II and cardiovascular panel III, immune response, inflammation, neuro‐exploratory and neurology (informed consent was given by all patients for the donation of blood samples for research purposes). Following quality control (where over 80% of patients had an existing protein recording) 476 unique proteins remained and were used for analysis. These proteins were then labelled whether they are differentially expressed in at least one model of cellular senescence according to external RNA‐sequencing (RNA‐seq). These data are from replicative and oncogenic models of senescence compared to non‐senescent controls, all within IMR‐90 cells.^36^, ^37^

### 2.3 Base demographics

All statistical testing and analytical pipelines were carried out in RStudio using the ‘finalfit’ and ‘stats’ packages. Demographical testing using chi‐squared was applied across gender, diabetes, CVD, hypercholesteremia and cause of CKD, with age, body mass index (BMI), creatinine, GFR, weight, diastolic blood pressure (BP) and systolic BP tested using ANOVA, all between mild CKD patients (stages 2 and 3a), moderate CKD patients (stage 3b) and severe CKD patients (stages 4 and 5) according to the KDIGO guidelines. This was done to ensure no confounding factors with overlapping proteomic metrics would cause bias in the extrapolation of our results.

### 2.4 Clustering pipeline for sendotype discovery

Clustering of CKD patients was carried out using the *k*‐means clustering algorithm from the ‘stats’ package. Prior to clustering, optimal features were selected by using principal component analysis (PCA) as a basic feature selection tool, using the ‘prcomp’ package. We selected the principal component (PC) that spanned the most class variance and ranked features according to the loading scores of each protein regardless of direction, focusing solely on magnitude. This was carried out with *n* = 500 bootstraps with the median loading scores being used as the ranking metric. Using a pre‐existing list of differentially expressed senescence transcripts from a previous study,^37^ we labelled each protein from the identified PC on whether its respective transcript was found to be significantly differentially expressed in multiple models of senescence.

With an optimal list of features, we then wanted to see the optimal number of features to use for clustering. We conducted a stepwise iterative *k*‐means clustering algorithm where the number of features used to cluster started with the single most significant protein and added proteins with forward feature selection until we attained the maximum number of proteins that met the significance threshold of effective clustering, a silhouette coefficient of. 5. To ensure that the number of clusters selected did not interfere with feature selection, this was applied to a range of clusters from 2 to 10.

The rationale for this methodology was to allow for the largest amount of biological information to be retained through protein numbers while still passing a statistical criterion. With our optimal proteins, we then carried out *k*‐means clustering and evaluated clustering using Calinski‒Harbarasz index, silhouette coefficients and the elbow method, which allowed us to see the intra‐ and inter‐cluster distances between data points of cluster assignments.

### 2.5 Differential expression analysis

Proteomic differential expression was determined using Welch's two‐tailed *t*‐test using the ‘stats’ package. Due to biological variation between a severe disease cluster cohort and a milder disease cluster cohort, we employed Welch's two‐tailed *t*‐test to account for unequal variance of proteomic recordings between these cohorts. Volcano plots were employed to visualise the overall differences in protein expression between cohorts, heatmaps were employed to visualise the individual patient proteomic differences between cohorts and violin boxplots were used to visualise both proteomic and clinical differences between cohorts for a single feature.

### 2.6 Proteomic and 1‐year follow‐up correlations

Patient eGFRs were calculated using the modification of diet in renal disease, which considers a patients age, sex, serum creatinine and race. Correlations between significantly identified proteins and 1‐year follow‐up recordings of both eGFR and creatinine were tested to see if the proteomic expression was directly proportional to worsening renal trajectories. This was done by building individual linear regression models fitting the data and using ordinary least squares test linearity between variables and denoted whether each datapoint was a mild, moderate or severe CKD patient. NPX protein expression was tested against log2(eGFR) and log2(creatinine), as NPX is already on a log2 base, adjusting follow‐up metrics was necessary to ensure the analysis was fair.

### 2.7 Pathway analysis

Pathway analysis was conducted in RStudio using the ‘pathfindR’ package. It used input such as associated gene symbol, adjusted *p*‐value and log2 FC, then filters this list of signatures down to significance by *p*‐value and finally returns all known enriched pathways containing these signatures, with enriched pathways retrieved from the Kyoto Encyclopedia of Genes and Genomes.

### 2.8 Validation using publicly available kidney organoid data

This study employed the use of publicly available data on the gene expression omnibus (GEO) to validate the proteins identified in our analysis. We retrieved transcriptomic data for six control kidney organoid models and six tumour necrosis factor‐alpha (TNF‐α) treated organoid models using the GSE259376 ID (establishment of epithelial inflammatory injury model using adult kidney organoids [RNA‐seq]). TNF‐α was used to induce tissue injury in said organoids, which we used as a model of kidney injury from CKD.^38^ We then performed differential expression analysis to investigate the differences in transcript expression between injured kidney organoids and healthy to see if the biological pathways they are involved in overlap with our CKD sendotype.

### 2.9 Validation using publicly available kidney biopsy data

This study employed the use of publicly available data on the GEO to validate the proteins and pathways identified in our analysis. We retrieved transcriptomic data for 18 healthy reference biopsies and 15 CKD biopsies with both cohorts being alive at time of sample collection (GSE183273).^39^ Transcriptomic data were quantified via single‐cell RNA‐seq (scRNA‐seq) and cell types were assigned to each biopsy‐derived cell based on transcriptomic profile similarity to reference cells using the Azimuth software. Cell‐type‐specific Pseudobulk analysis was performed, wherein each patient had their expression quantified for immune cells, endothelial cells and epithelial cells by summing the expression counts for individual genes within a distinct cell type. This gave each patient three unique datapoints for each cell type, which were then converted from counts per million to transcripts per million. Three CKD patients were removed from the analysis (IDs: 27–10 039, 28–10 051 and 31–10 000) due to very low overall gene expression levels within specific cell types, resulting in the final cohort size of 12 CKD biopsies and 18 healthy reference biopsies, with each biopsy having three recordings for different cell types.

## 3 RESULTS

### 3.1 Demographics of CKD patients

**FIGURE 1:**
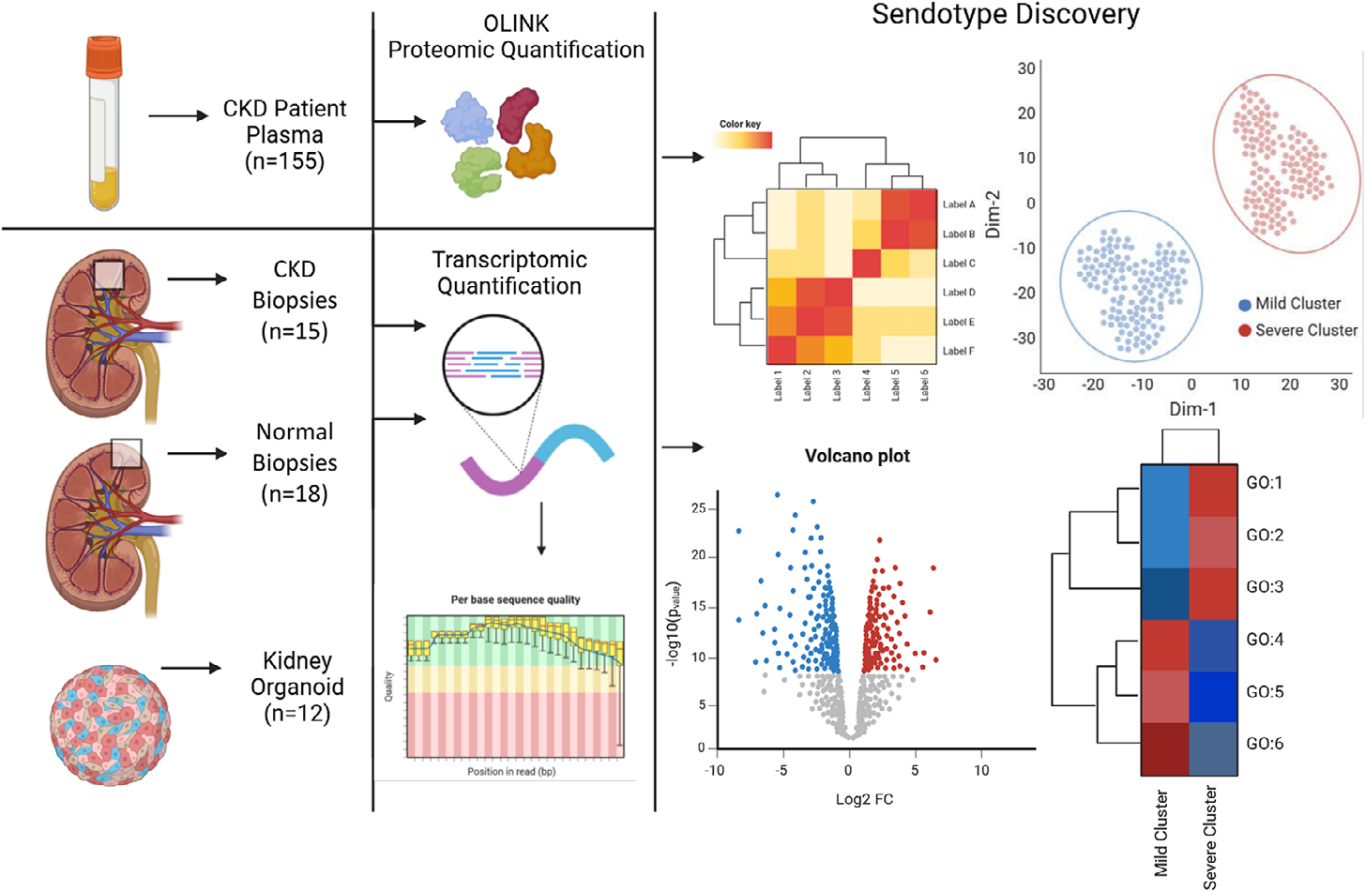
Graphical abstract of the chronic kidney disease (CKD) sendotype discovery pipeline demonstrating the use of multiple omic datasets from patient plasma, patient biopsies and kidney organoid models for both clustering and validation.

Baseline demographics were generated for our CKD cohort, prior to our sendotype discovery pipeline (Figure 1). Gender, BMI, systolic BP, diastolic BP, hypocholesteraemia, CVD and cause of CKD were found to be not significant between our cohorts (Table 1). We did however find age (*p *= .014), weight (*p *= .048), diabetes (*p *= .012), creatinine (*p *< .001) and eGFR (*p *< .001) to be significantly different between CKD subgroups. We expected both creatinine and eGFR to be significant as we used three distinct groups of mild, moderate and severe CKD by splitting the clinical classification according to the KDIGO into three distinct groups. However, age being identified as significant could suggest that the underlying mechanism of senescence is the driver of this significance and not biological age. Although both weight and diabetes were both identified as significant between our CKD cohorts, we believe due to their nature of being risk factors for CKD, this was unavoidable and does not affect downstream analysis.

**TABLE 1:** Base demographics for patients.

Label	Levels	Mild (*n* = 40)	Moderate (*n* = 23)	Severe (*n* = 16)	*p*‐Value
Age (years)	Mean (SD)	52.2 (17.1)	59.3 (16.4)	66.2 (13.3)	.014
Gender	Female	18 (45.0)	10 (43.5)	4 (25.0)	.365
	Male	22 (55.0)	13 (56.5)	12 (75.0)	
Creatinine (µmol/L)	Mean (SD)	95.8 (25.9)	152.3 (26.0)	222.4 (68.3)	<.001
GFR (MDRD)	Mean (SD)	55.7 (6.5)	37.5 (6.1)	26.5 (9.8)	<.001
Weight (kg)	Mean (SD)	79.4 (17.3)	91.0 (21.3)	86.7 (14.3)	.048
BMI (kg/m^2^)	Mean (SD)	28.6 (6.0)	31.6 (6.7)	29.5 (4.5)	.161
Systolic (mmHg)	Mean (SD)	140.7 (18.0)	144.5 (19.1)	146.6 (17.9)	.492
Diastolic (mmHg)	Mean (SD)	82.0 (11.2)	79.9 (9.5)	80.5 (9.8)	.730
Diabetes	No	36 (90.0)	14 (60.9)	10 (62.5)	.012
	Yes	4 (10.0)	9 (39.1)	6 (37.5)	
Hypercholesterolaemia	No	29 (72.5)	11 (47.8)	8 (50.0)	.095
	Yes	11 (27.5)	12 (52.2)	8 (50.0)	
CVD	CVD present	5 (12.5)	4 (17.4)	4 (25.0)	.517
	No CVD	35 (87.5)	19 (82.6)	12 (75.0)	
Cause of CKD	Acute kidney injury	5 (12.5)	1 (4.3)	1 (6.2)	.094
	Autosomal dominant polycystic kidney disease	5 (12.5)	0 (0)	0 (0)	
	Chronic pyelonephritis	1 (2.5)	0 (0)	0 (0)	
	Chronic renal impairment	1 (2.5)	4 (17.4)	3 (18.8)	
	Glomerulonephritis	9 (22.5)	1 (4.3)	2 (12.5)	
	Haematuria	2 (5.0)	0 (0)	0 (0)	
	Heart failure	1 (2.5)	0 (0)	0 (0)	
	Hyperplasia of renal artery	1 (2.5)	0 (0)	0 (0)	
	Hypertensive renal disease	2 (5.0)	1 (4.3)	1 (6.2)	
	IgA nephropathy	7 (17.5)	7 (30.4)	1 (6.2)	
	Proteinuria	1 (2.5)	0 (0)	0 (0)	
	Unknown	4 (10.0)	3 (13.0)	2 (12.5)	
	Urinary tract infection	1 (2.5)	0 (0)	0 (0)	
	Microscopic polyangiitis	0 (0)	1 (4.3)	0 (0)	
	Microscopic polyarteritis nodosa	0 (0)	1 (4.3)	0 (0)	
	Renal vascular disease	0 (0)	1 (4.3)	2 (12.5)	
	Tubular necrosis	0 (0)	1 (4.3)	0 (0)	
	Urolithiasis (kidney stones)	0 (0)	1 (4.3)	0 (0)	
	Vesicoureteric reflux	0 (0)	1 (4.3)	0 (0)	
	Cardio‐renal syndrome	0 (0)	0 (0)	1 (6.2)	
	Diabetic nephropathy	0 (0)	0 (0)	1 (6.2)	
	Drug‐induced nephritis	0 (0)	0 (0)	1 (6.2)	
	Tubulo interstitial nephritis	0 (0)	0 (0)	1 (6.2)	

Abbreviations: BMI, body mass index; CKD, chronic kidney disease; CVD, cardiovascular disease; GFR, glomerular filtration rate; MDRD, modification of diet in renal disease; SD, standard deviation.

### 3.2 PCA feature selection identifies optimal senescence panel

**FIGURE 2:**
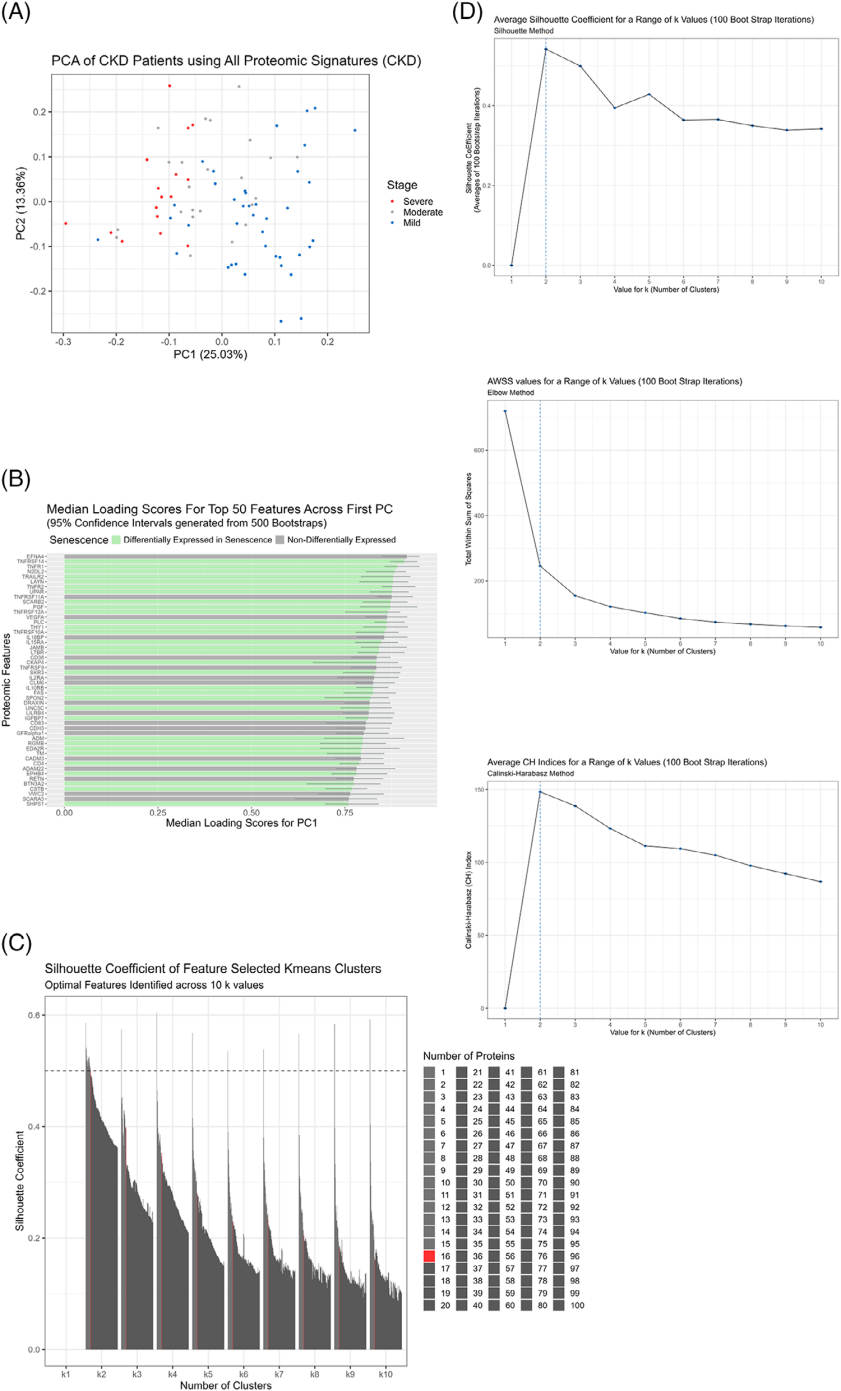
Principal component analysis (PCA) feature selection identifies optimal senescence panel. (A) PCA of chronic kidney disease (CKD) patients and their severity status using all recorded plasma proteins. (B) Median loading scores of each protein across multiple bootstraps of PC1, labelled green if those proteins corresponding transcript is differentially expressed in senescence. (C) Silhouette coefficient plot of multiple different protein sets used to cluster CKD patients across 10 ranges of cluster numbers, identifying 16 proteins as most optimal at *k* = 2. (D) Optimal cluster number validation using the Calinski‒Harbarasz index, elbow method and silhouette method to measure inter‐ and intra‐cluster distances.

Prior to *k*‐means clustering, we wanted to see the overall proteomic differences between our CKD patients related to disease severity and then identify a smaller subset that best characterises the patients. Using PCA, we identified PC1, which was responsible for the majority of variance between CKD patients with varying disease severity (Figure 2A). Using this PC, we ranked each protein within the dataset according to the magnitude of their loading scores, which created a descending list of proteins and how important they were to disease severity. This was carried out 500 times with bootstrap sampling and the median loading scores were plotted for each protein from largest to smallest for the top 50 proteins. We found that the most important proteins associated with CKD severity were differentially expressed in senescence, 64% of the top 50 proteins (Figure 2B). We then identified 16 proteins as the largest number of proteins that maintained a silhouette coefficient at or above. 5 (PGF, TNFRSF10A, TNFRSF11A, TRAILR2, TNFRSF14, TNFR2, PLC, UPAR, TNFR1, VEGFA, EFNA4, SCARB2, LAYN, THY1, TNFRSF12A and N2DL2) to be the optimal number of proteins to use for *k*‐means clustering. Of these 16, 13 are differentially expressed in senescence coloured in green according to whether their corresponding transcript was differentially expressed in either oncogenic or replicative senescence models from a separate RNA‐seq experiment (Figure 2B,C). Lastly, we assessed the clustering quality of the *k*‐means clusters with these 16 proteins by employing three‐independent clustering tests, Calinski‒Harbarasz, silhouette method and elbow plots, all of which demonstrated that *k* = 2 was the optimal number of clusters and that the clusters were of good quality according to their inter‐ and intra‐cluster distances (Figure 2D).

### 3.3 Upregulated senescent proteins cluster severe CKD patients

**FIGURE 3:**
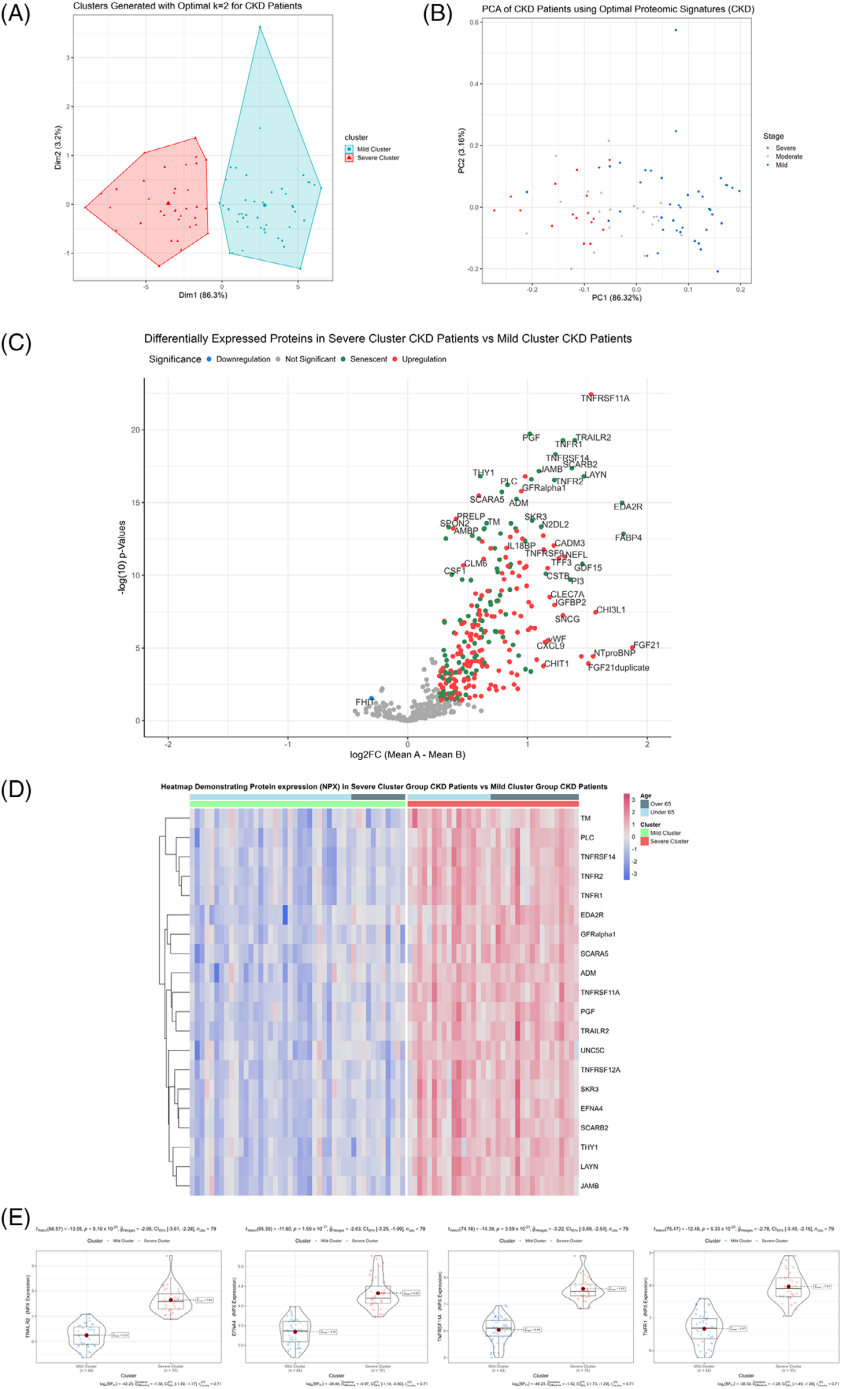
Upregulated senescent proteins cluster severe chronic kidney disease (CKD) patients. (A) *k*‐Means of CKD patients using the optimal panel of proteins to cluster patients into mild and severe cohorts. (B) Principal component analysis (PCA) of CKD patients using the optimal panel of proteins to show better separation of severity when using the optimal panel. (C) Volcano plot showing differential expression of proteins between severe and mild CKD clusters with the *x*‐axis showing log2 FC and the *y*‐axis showing ‒log10(*p*‐value). (D) Heatmap of the top set of differentially expressed proteins between severe and mild CKD clusters, with rows representing proteins and columns representing patients. (E) Violin‐boxplots showing differential expression of proteins using individual expression values of patients within severe and mild cluster cohorts.

After identifying the best subset of proteins to cluster patients into potential sendotype groups, we then carried out *k*‐means clustering of all CKD patients using the optimal 16 proteins (Figure 3A) and looked at the quality of cluster assignments by comparing the *k*‐means with a follow‐up PCA that only used these 16 proteins (Figure 3B). Remarkably, we found that the mild cluster contained no severe CKD patients and was primarily mild patients with a few moderate patients. The severe cluster contained six mild patients only, with the remainder being predominantly severe and moderate patients. The next step was to look at all differentially expressed proteins between the cluster cohorts, which when visualised on heatmaps and volcano plots we can see that most proteins that are significantly differentially expressed are senescent proteins and that the direction of regulation of the majority of these proteins are upregulation (Figure 3C,D). Lastly, using violin‐boxplots, we visualised the most significant proteins between our cluster groups according to both *p*‐value and log2 FC, which returned EFNA4, TRAILR2, TNFRSF11A and TNFR1 as being the most significant (*p*‐values, etc.). We then queried all statistically differentially expressed proteins between our two sendotype groups to identify potential drivers of the sendotype, which revealed cytokine‒cytokine receptor interactions, viral protein interaction with cytokine‒cytokine receptor, chemokine signalling, NF‐κB, TNF and apoptosis to be the most enriched yet biologically relevant pathways (Table 2).

**TABLE 2:** Enriched pathways between chronic kidney disease sendotypes.

ID	Term description	Fold enrichment	Occurrence	Support	Lowest *p*‐value
hsa04060	Cytokine‒cytokine receptor interaction	18.9625588	10	.215236	2.80 × 10^−21^
hsa04061	Viral protein interaction with cytokine and cytokine receptor	27.7633636	10	.09114	1.00 × 10^−20^
hsa04062	Chemokine signalling pathway	7.8006897	10	.005129	1.20 × 10^−17^
hsa04064	NF‐κB signalling pathway	10.2827273	10	.043339	2.00 × 10^−10^
hsa05205	Proteoglycans in cancer	6.1912842	10	.07634	6.70 × 10^−10^
hsa05163	Human cytomegalovirus infection	5.0018492	10	.056424	1.20 × 10^−08^
hsa04668	TNF signalling pathway	11.6469703	10	.052497	3.00 × 10^−08^
hsa04512	Extra cellular matrix (ECM)‒receptor interaction	7.5406667	10	.015348	5.20 × 10^−08^
hsa04210	Apoptosis	5.9336393	10	.112847	7.70 × 10^−08^

Abbreviations: NF‐κB, nuclear factor‐kappa B; TNF, tumour necrosis factor.

### 3.4 Sendotype patients have deteriorated renal function indicators

**FIGURE 4:**
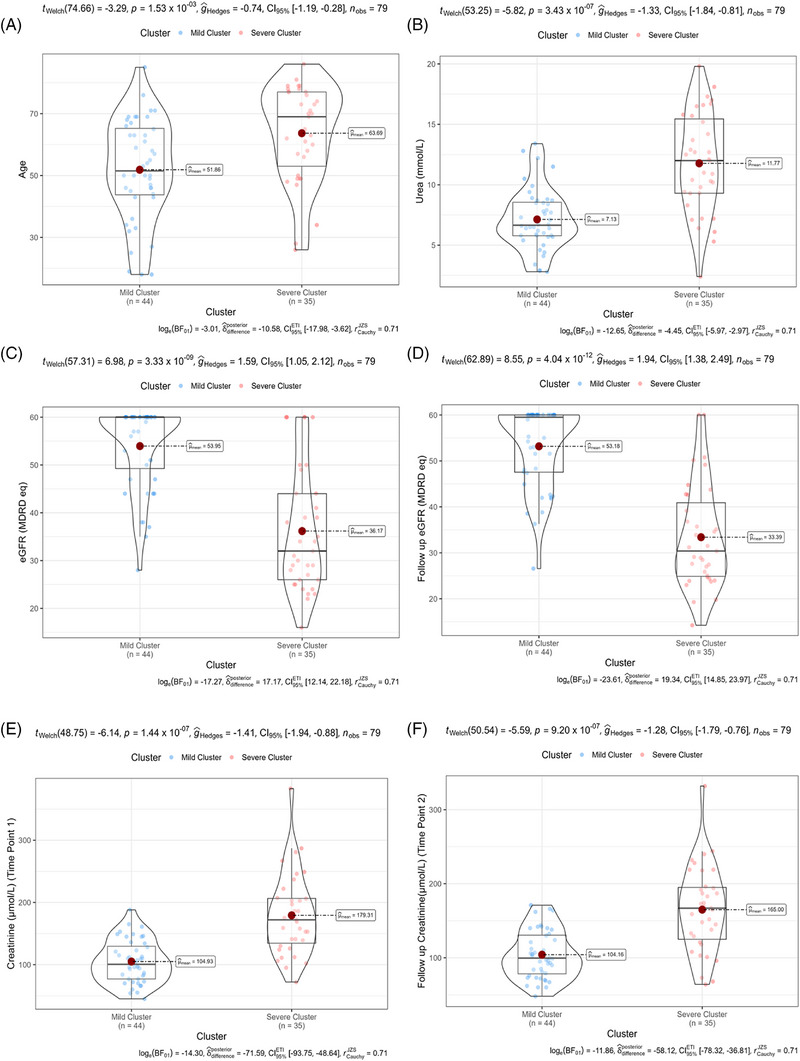
Sendotype patients have deteriorated renal function indicators. (A) Violin‐boxplot comparing the individual ages of chronic kidney disease (CKD) patients within severe and mild clusters (age measured in years). (B) Violin‐boxplot comparing the individual urea levels of CKD patients within severe and mild clusters (urea measured in mmol/L). (C) Violin‐boxplot comparing the individual estimated glomerular filtration rate (eGFR) recordings of CKD patients within severe and mild clusters (eGFR measured by modification of diet in renal disease [MDRD] equation). (D) Violin‐boxplot comparing the individual follow‐up eGFR recordings of CKD patients within severe and mild clusters (eGFR measured by MDRD equation). (E) Violin‐boxplot comparing the individual creatinine levels of CKD patients within severe and mild clusters (creatinine measured in µmol/L). (F) Violin‐boxplot comparing the individual follow‐up creatinine levels of CKD patients within severe and mild clusters (creatinine measured in µmol/L).

With significant proteins identified between our sendotype patients, we then wanted to identify potential clinical differences that could also be characterised by these sendotypes. We visualised clinical differences between our severe and mild clustered sendotype patients and identified that our severe sendotype had more biologically aged patients (Figure 4A), higher levels of urea (Figure 4B), had reduced renal function according to both lower eGFR (Figure 4C) and lower 1‐year follow‐up eGFR (Figure 4D) and had higher levels of both creatinine (Figure 4E) and 1‐year follow‐up creatinine (Figure 4F). This also confirmed that the clustering and identification of our sendotypes was successful as with just using 16 proteins, patients were stratified using clustering into two unique sendotypes with differing disease states, differing proteomic measurements and differing clinical symptomologies.

### 3.5 Sendotype signatures correlate with worsening renal trajectories

**FIGURE 5:**
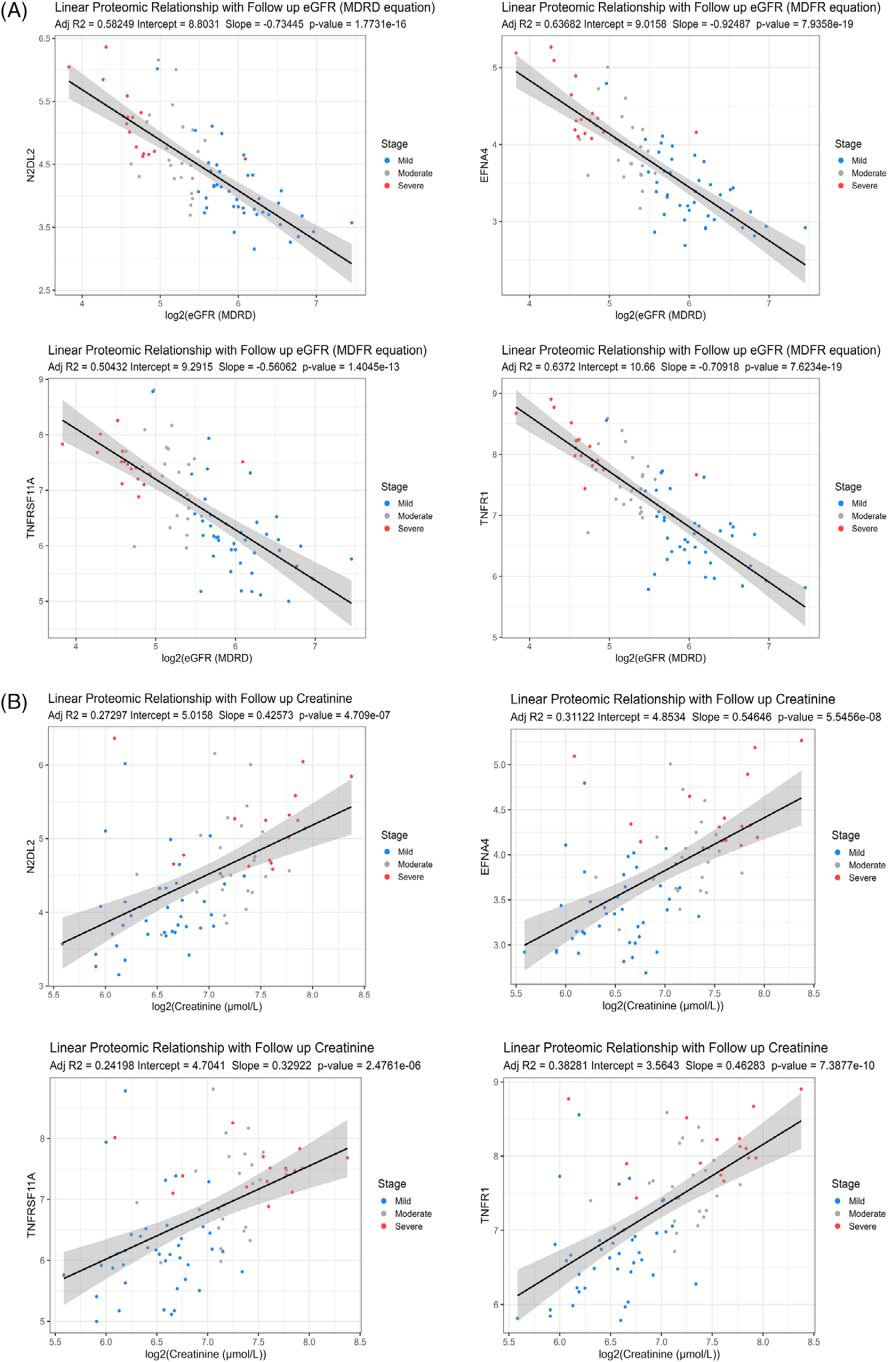
Sendotype signatures correlate with worsening renal trajectories. (A) Linear‐regression models fitting N2DL2, EFNA4, TNFRSF11A and TNFR1 NPX levels (*y*‐axis) against log2(eGFR follow‐up recordings) (*x*‐axis) in individual models, clinically labelled from red to blue according to chronic kidney disease (CKD) patient severity. (B) Linear‐regression models fitting N2DL2, EFNA4, TNFRSF11A and TNFR1 NPX levels (*y*‐axis) against log2(creatinine follow‐up recordings) (*x*‐axis) in individual models, clinically labelled from red to blue according to CKD patient severity. eGFR, estimated glomerular filtration rate.

As eGFR is the current gold standard of testing renal decline and is based off creatinine levels, we wanted to see if the identified sendotype proteomics followed the same trends as these clinical variables at 1‐year follow‐ups. We used our 16 identified sendotype proteins that clustered the CKD cohorts and built linear regression models of each while applying log2 to both eGFR and creatinine (best four subset was selected for display). We found that all proteins for eGFR had an *R* ^2^ value above. 5, *p*‐value less than. 0001, and followed the same inversely proportional trend (Figure 5A). As protein expression levels decreased, the overall renal function according to eGFR increased, suggesting that these proteins are indeed associated with worsening renal function. When looking at the proteins plotted with creatinine, they had slightly less strong correlation but still had statistically powerful *p*‐values less than. 0001.

### 3.6 Kidney injury organoids validate sendotype pathways

**FIGURE 6:**
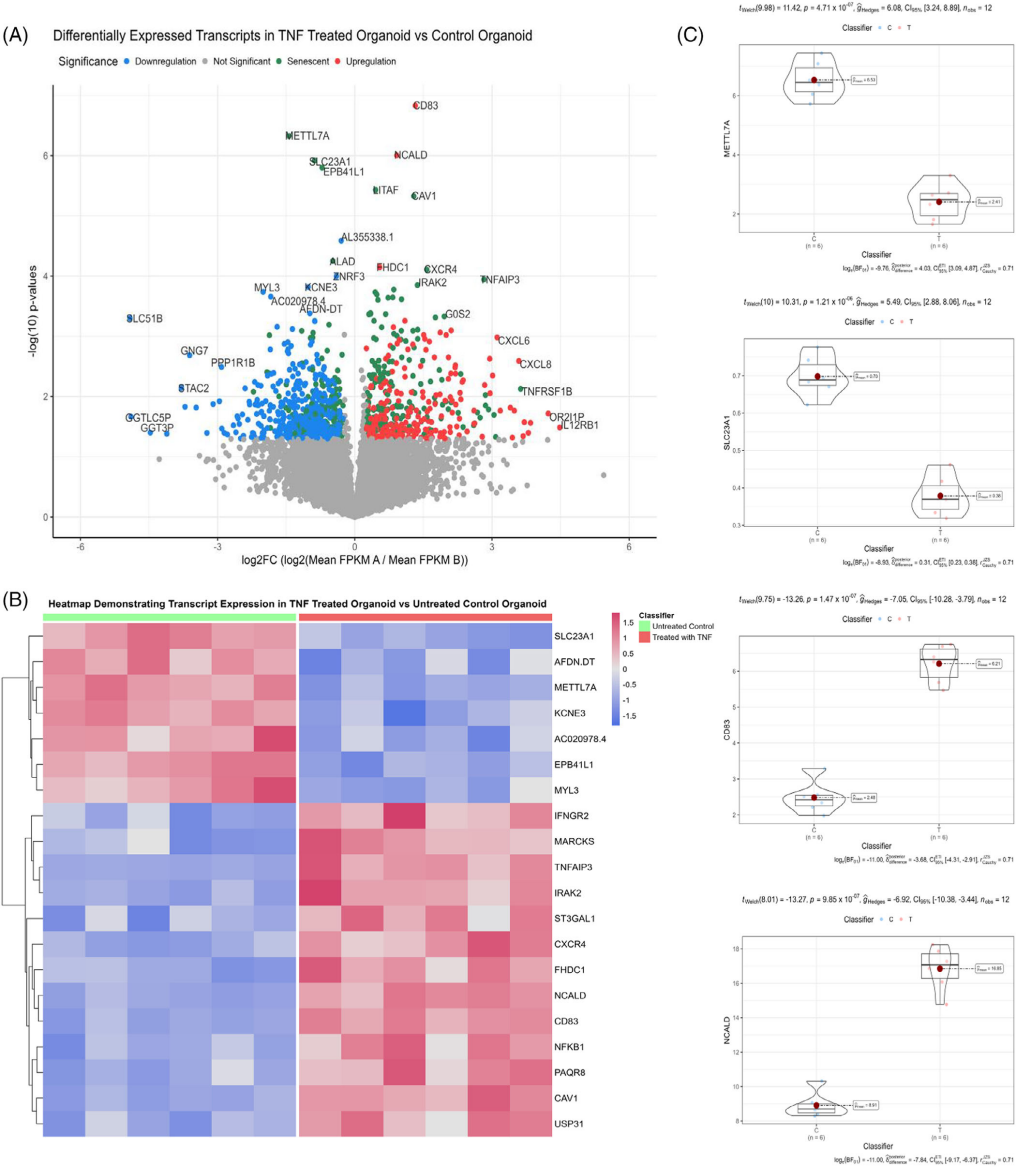
Kidney injury organoids validate sendotype pathways. (A) Volcano plot showing differential expression of transcripts between injured and non‐injured kidney organoids with the *x*‐axis showing log2 FC and the *y*‐axis showing ‒log10(*p*‐value). (B) Heatmap of the top set of differentially expressed transcripts between injured and non‐injured kidney organoids, with rows representing transcripts and columns representing organoids. (C) Violin‐boxplots showing differential expression of transcripts using individual expression values of kidney organoids within injured and non‐injured cohorts.

We then wanted to validate the biologically important pathways within our sendotype patients in renal‐specific cells, proving that these pathways are indeed important to CKD pathophysiology. To do this, we employed the use of the GEO and selected the ‘GSE259376’ study for differential expression validation, which used multiple kidney organoid models to investigate kidney injury. We first visualised the transcriptomic differential expression differences between healthy and injured kidney organoids using volcano plots and heatmaps, which showed that out of the top 10 most significant transcripts, seven are differentially expressed in senescence (Figure 6A,B). Using violin‐boxplots we then focused on the individual transcript expression between the two groups and found that CD83 and NCALD are the most significant upregulated transcripts and METTL7A alongside SLC23A1 are the most significant downregulated transcripts in the kidney injury organoid model compared to the healthy model. We then queried all significant transcripts between kidney injury organoids and healthy kidney organoids to identify significantly enriched signalling pathways indicative of renal tissue damage. We found that TNF signalling, NF‐κB signalling, cytokine‒cytokine receptor interactions, cellular senescence, apoptosis and MAPK to be significant biologically enriched pathways between the organoid groups, with most of these pathways also identified as significant in our prior proteomic clustering analysis (Table 3).

**TABLE 3:** Enriched pathways between kidney organoids.

ID	Term description	Fold enrichment	Occurrence	Support	Lowest *p*‐value
hsa04060	Cytokine‒cytokine receptor interaction	4.3707494	10	.0121212	6.90 × 10^−06^
hsa04218	Cellular senescence	3.0038814	10	.0422965	4.40 × 10^−06^
hsa04064	NF‐κB signalling pathway	6.5618004	10	.1502567	2.00 × 10^−10^
hsa04668	TNF signalling pathway	8.0713585	10	.1441558	1.10 × 10^−13^
hsa04512	ECM‒receptor interaction	3.6393179	10	.0196087	3.30 × 10^−04^
hsa04210	Apoptosis	5.9336393	10	.1128472	7.7 × 10^−08^
hsa04010	MAPK signalling pathway	2.0586041	10	.0060606	9.30 × 10^−04^

Abbreviations: NF‐κB, nuclear factor‐kappa B; TNF, tumour necrosis factor.

### 3.7 Transcriptomic validation using publicly available kidney biopsy datasets

**FIGURE 7:**
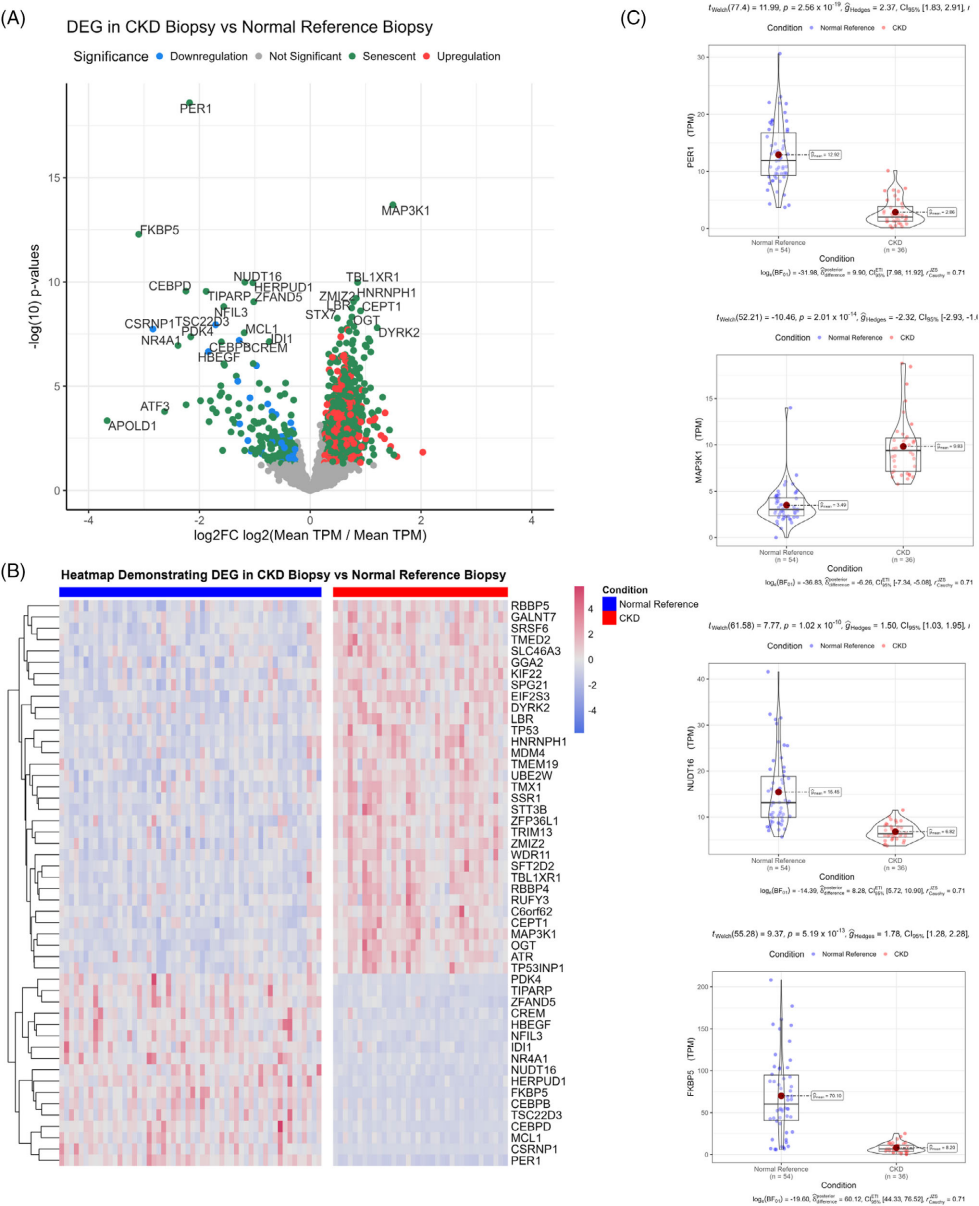
Chronic kidney disease (CKD) biopsies validate sendotype pathways. (A) Volcano plot showing differential expression of transcripts between CKD biopsies and normal reference biopsies with the *x*‐axis showing log2 FC and the *y*‐axis showing ‒log10(*p*‐value). (B) Heatmap of the top set of differentially expressed transcripts between CKD biopsies and normal reference biopsies, with rows representing transcripts and columns representing patients. (C) Violin‐boxplots showing differential expression of transcripts using individual expression values of CKD biopsies and normal reference biopsies.

Next, we wanted to validate the biologically important pathways within our sendotype patients in biopsies taken from other CKD patients. To do this, we employed the use of the GEO and selected the ‘GSE183273’ study for differential expression validation, which used multiple kidney biopsies of live non‐CKD reference patients compared to live CKD patients. We first visualised the transcriptomic differential expression differences between CKD and normal reference biopsies using volcano plots and heatmaps, which showed that there are several senescent transcripts that are differentially expressed in CKD biopsies compared to normal reference biopsies (Figure 7A). Two distinct hierarchical clusters of upregulation and downregulation were identified (Figure 7B) between CKD biopsies and normal reference biopsies. Using violin‐boxplots, we then focused on the individual transcript expression between the two cohorts. We found that PER1, MAP3K1, NUDT16 and FKBP5 were the most differentially expressed transcripts between CKD and normal reference biopsies (Figure 7C). We then queried all significant transcripts between CKD biopsies and normal reference biopsies to identify significantly enriched signalling pathways between cohorts. We found that MAPK signalling, NF‐κB, cellular senescence, cell cycle, TNF signalling, apoptosis and transforming growth factor‐beta signalling to be the most significant biologically relevant enriched pathways between the CKD biopsies and reference biopsies, with most of these pathways also identified as significant in our prior proteomic clustering analysis, as well as the organoid analysis (Table 4).

**TABLE 4:** Enriched pathways between chronic kidney disease and normal reference biopsies.

ID	Term description	Fold enrichment	Occurrence	Support	Lowest *p*‐value
hsa04010	MAPK signalling pathway	2.3319726	10	.1600000	3.2 × 10^−18^
hsa04064	NF‐κB signalling pathway	2.6711686	10	.1012665	8.8 × 10^−17^
hsa04218	Cellular senescence	2.9696957	10	.1852453	1.0 × 10^−16^
hsa04110	Cell cycle	2.7790681	10	.2244956	7.2 × 10^−16^
hsa04668	TNF signalling pathway	3.2139662	10	.1281566	5.5 × 10^−15^
hsa04210	Apoptosis	2.4772430	10	.0813908	6.0 × 10^−12^
hsa04350	TGF‐β signalling pathway	1.3432162	10	.0731506	1.2 × 10^−11^

Abbreviations: NF‐κB, nuclear factor‐kappa B; TGF‐β, transforming growth factor‐beta; TNF, tumour necrosis factor.

## 4 DISCUSSION

Our findings have demonstrated that using a small panel of predominantly senescent proteins, CKD patients can be clustered separately into distinct sendotypes according to disease severity. The clustered CKD patients not only show differing proteomic expressional patterns but also have multiple differing clinical symptomologies consistent with disease severity such as eGFR, creatinine and urea, which are consistent at 1‐year follow‐ups for both creatinine and eGFR. We also found that when testing the correlation of these sendotype proteins with 1‐year follow‐up clinical metrics they share a trajectory of inversely proportional correlations, suggesting these proteins are highly involved in CKD symptomology and pathophysiology.

Of the 16 proteins identified for optimal patient clustering, the top four include EFNA4, TNFRSF14, TNFR1 and N2DL2, with the latter three being differentially expressed in senescence according to our transcriptomic senescent list of signatures. EFNA4 is a cell surface bound ligand for Eph receptors, a family of tyrosine kinases responsible for migration, repulsion and adhesion during neuronal, vascular and epithelial development.^40^ Recent literature has demonstrated EFNA4's use as a potential prognostic marker to stratify patients according to risk of ESRD.^41^, ^42^

The TNFR1 and TNFRSF14 genes encode members of the TNF receptor superfamily of proteins.^43^ Binding of membrane‐bound TNF‐α to these receptor proteins induces receptor trimerisation and activation, which plays a role in cell survival, apoptosis and inflammation.^43^ The TNF‐α‒TNFR1 signalling pathway these proteins are involved in are also implicated in the inducement of excessive senescence.^44^ These proteins are also involved in kidney disease pathology via renal fibrosis,^45^ IgA nephropathy^46^ and have been shown to stratify patient risk of ESRD.^41^, ^42^

N2DL2 also known as ULBP2 is a member of the MHC‐1 class superfamily and is responsible for mediating natural killer cell cytotoxicity.^43^ Although little is known about N2DL2 in the context of CKD, studies have demonstrated its upregulation in multiple models of senescence including both replicative, DNA‐damage induced and oncogenic models of senescence.^47^, ^48^

These overexpressed senescent proteins in our severe sendotype patients play vital roles in many signalling pathways such as NF‐κB, cytokine‒cytokine interactions, apoptosis, MAPK and TNF signalling, which are all identified as significantly enriched pathways in the transcriptomic validation datasets. The canonical NF‐κB signalling pathway is activated in the presence of various pro‐inflammatory cytokines such as TNF's and IL's, which activate the IkB kinase (IKK) complex.^49^ Once activated IKK‐β phosphorylates IkB, which results in ubiquitination and degradation and frees many NF‐κB dimers. These dimers then translocate to the nucleus, bind to the promotor regions of target genes and cause the transcription of these genes and the subsequent expression of various inflammatory signatures.^49^, ^50^, ^51^ NF‐κB plays a pivotal role in the pathogenesis of CKD^52^ and has been shown to be one of the main SASP pathways.^53^ However, little research is available on the involvement of NF‐κB within the context of both CKD and senescence combined. Our preliminary results suggest that in patients with higher levels of senescence and SASP expression, various signalling pathways such as NF‐κB, apoptosis, chemokine and TNFs become more enriched, which results in a more pronounced inflammatory cascade, which correlates with the advances of the pathogenesis of CKD. This would suggest that overexpression of these senescence signatures in CKD patients creates a distinct sendotype wherein patients have more pronounced inflammatory markers, worsening renal functions, worsening renal trajectories and various biological signalling events causing worsened health. These findings have been shown in patient plasma proteomics with the biological signalling events been validated in kidney tissues for both CKD biopsies and organoid models via transcriptomic analysis. This suggests that CKD needs to be endotyped to better stratify patients. In doing so, it could prove beneficial by identifying potential new drug targets and further the understanding of CKD pathophysiology.

## 5 LIMITATIONS

The research in question while methodologically correct does however have its short comings. The effect of clinical drivers such as weight and comorbidities were not fully removed from the study, which would have been a useful investigative tool to examine pathobiology independently from these factors. Another limitation is the small sample sizes, as the CKD proteomic study consisted of 79 participants, with further validation in larger cohorts being required for robustness.

Lastly, one of the major limitations within the study was that crucial ECR data such as proteinuria measurements were skewed and had to be left out from the study, which could have proved useful throughout the analysis.

## AUTHOR CONTRIBUTIONS

All the authors warrant that this article is the authors’ original work, has not received prior publication and is not under consideration for publication elsewhere. Thomas McLarnon and Taranjit Singh Rai wrote the scientific paper, with scientific and clinical input from Frank McCarroll, Ying Kuan, Elaine K. Murray, Steven Watterson, David S. Gibson and Shu‐Dong Zhang. Taranjit Singh Rai, Steven Watterson and Thomas McLarnon conceived the idea and designed the research. Anthony J. Bjourson and Taranjit Singh Rai secured funding. Thomas McLarnon performed analysis under the supervision of Taranjit Singh Rai and Steven Watterson, based on previous work by Eamonn Cooper. Sample collection and processing was carried out by Sean McCallion and Andrew R. English.

## CONFLICT OF INTEREST STATEMENT

The authors declare they have no conflicts of interest.

## ETHICS STATEMENT

The study was conducted in accordance with the Declaration of Helsinki and approved by the Institutional Review Board, Manchester Northwest Research Ethics Committee (Research Protocol version 7.0 [01/08/2019]).

## INFORMED CONSENT STATEMENT

Informed consent was obtained from all subjects involved in the study.

## Data Availability

The raw data supporting the conclusions of this article will be made available by the authors upon request.

## References

[1] Kovesdy CP . Epidemiology of chronic kidney disease: an update 2022. Kidney Int Suppl (2011). 2022;12:7‐11. 35529086 10.1016/j.kisu.2021.11.003 PMC9073222

[2] Bikbov B , Purcell CA , Levey AS , et al. Global, regional, and national burden of chronic kidney disease, 1990–2017: a systematic analysis for the Global Burden of Disease Study 2017. The Lancet. 2020;395:709‐733. 10.1016/S0140-6736(20)30045-3 PMC7049905 32061315

[3] Levey AS , de Jong PE , Coresh J , et al. The definition, classification, and prognosis of chronic kidney disease: a KDIGO Controversies Conference report. Kidney Int. 2011;80:17‐28. 21150873 10.1038/ki.2010.483

[4] Webster AC , Nagler EV , Morton RL , Masson P . Chronic kidney disease. Lancet. 2017;389:1238‐1252. 27887750 10.1016/S0140-6736(16)32064-5

[5] Mallappallil M , Friedman EA , Delano BG , McFarlane SI , Salifu MO . Chronic kidney disease in the elderly: evaluation and management. Clin Pract. 2014;11:525‐535. 10.2217/cpr.14.46 PMC4291282 25589951

[6] Lindeman RD , Tobin J , Shock NW . Longitudinal studies on the rate of decline in renal function with age. J Am Geriatr Soc. 1985;33:278‐285. 3989190 10.1111/j.1532-5415.1985.tb07117.x

[7] Figuer A , Bodega G , Tato P , et al. Premature aging in chronic kidney disease: the outcome of persistent inflammation beyond the bounds. Int J Environ Res Public Health. 2021;18:8044. 34360333 10.3390/ijerph18158044 PMC8345753

[8] Abdel‐Rahman EM , Okusa MD . Effects of aging on renal function and regenerative capacity. Nephron Clin Pract. 2014;127:15‐20. 25343814 10.1159/000363708

[9] O'Sullivan ED , Hughes J , Ferenbach DA . Renal aging: causes and consequences. J Am Soc Nephrol. 2017;28:407‐420. 28143966 10.1681/ASN.2015121308 PMC5280008

[10] Rex N , Melk A , Schmitt R . Cellular senescence and kidney aging. Clin Sci. 2023;137:1805‐1821. 10.1042/CS20230140 PMC10739085 38126209

[11] Zhao J , Qiao X , Mao J , Liu F , Fu H . The interaction between cellular senescence and chronic kidney disease as a therapeutic opportunity. Front Pharmacol. 2022;13:974361. 36091755 10.3389/fphar.2022.974361 PMC9459105

[12] Chi M , Tian Z , Ma K , et al. The diseased kidney: aging and senescent immunology. Immunity Ageing. 2022;19(1):58. 36384564 10.1186/s12979-022-00313-9 PMC9666969

[13] Hayflick L , Moorhead PS . The serial cultivation of human diploid cell strains. Exp Cell Res. 1961;25:585‐621. 13905658 10.1016/0014-4827(61)90192-6

[14] Mitry MA , Laurent D , Keith BL , et al. Accelerated cardiomyocyte senescence contributes to late‐onset doxorubicin‐induced cardiotoxicity. Am J Physiol Cell Physiol. 2020;318:C380‐C391. 31913702 10.1152/ajpcell.00073.2019 PMC7052608

[15] Barnes PJ , Baker J , Donnelly LE . Cellular senescence as a mechanism and target in chronic lung diseases. Am J Respir Crit Care Med. 2019;200:556‐564. 30860857 10.1164/rccm.201810-1975TR

[16] Schuliga M , Read J , Knight DA . Ageing mechanisms that contribute to tissue remodeling in lung disease. Ageing Res Rev. 2021;70:101405. 34242806 10.1016/j.arr.2021.101405

[17] Lee S , Yu Y , Trimpert J , et al. Virus‐induced senescence is a driver and therapeutic target in COVID‐19. Nature. 2021;599:283‐289. 34517409 10.1038/s41586-021-03995-1

[18] Coppé J , Patil CK , Rodier F , et al. Senescence‐associated secretory phenotypes reveal cell‐nonautonomous functions of oncogenic RAS and the p53 tumor suppressor. PLoS Biol. 2008;6:e301. 19053174 10.1371/journal.pbio.0060301 PMC2592359

[19] Tan H , Xu J , Liu Y . Ageing, cellular senescence and chronic kidney disease: experimental evidence. Curr Opin Nephrol Hypertens. 2022;31:235‐243. 35142744 10.1097/MNH.0000000000000782 PMC9035037

[20] Kirkland JL , Tchkonia T . Cellular senescence: a translational perspective. EBioMedicine. 2017;21:21‐28. 28416161 10.1016/j.ebiom.2017.04.013 PMC5514381

[21] Prata LGPL , Ovsyannikova IG , Tchkonia T , Kirkland JL . Senescent cell clearance by the immune system: emerging therapeutic opportunities. Semin Immunol. 2018;40:101275. 31088710 10.1016/j.smim.2019.04.003 PMC7061456

[22] Song P , An J , Zou M . Immune clearance of senescent cells to combat ageing and chronic diseases. Cells. 2020;9:671. 32164335 10.3390/cells9030671 PMC7140645

[23] Annuk M , Zilmer M , Lind L , Linde T , Fellström B . Oxidative stress and endothelial function in chronic renal failure. J Am Soc Nephrol. 2001;12:2747‐2752. 11729244 10.1681/ASN.V12122747

[24] Irazabal MV , Torres VE . Reactive oxygen species and redox signaling in chronic kidney disease. Cells. 2020;9:1342. 32481548 10.3390/cells9061342 PMC7349188

[25] Nakagawa K , Itoya M , Takemoto N , et al. Indoxyl sulfate induces ROS production via the aryl hydrocarbon receptor‐NADPH oxidase pathway and inactivates NO in vascular tissues. Life Sci. 2021;265:118807. 33232689 10.1016/j.lfs.2020.118807

[26] Taki K , Nakamura S , Miglinas M , Enomoto A , Niwa T . Accumulation of indoxyl sulfate in OAT1/3‐positive tubular cells in kidneys of patients with chronic renal failure. J Ren Nutr. 2006;16:199‐203. 16825019 10.1053/j.jrn.2006.04.020

[27] Muteliefu G , Enomoto A , Jiang P , Takahashi M , Niwa T . Indoxyl sulphate induces oxidative stress and the expression of osteoblast‐specific proteins in vascular smooth muscle cells. Nephrol Dial Transplant. 2009;24:2051‐2058. 19164326 10.1093/ndt/gfn757

[28] Zhang J , Wang X , Vikash V , et al. ROS and ROS‐mediated cellular signaling. Oxid Med Cell Longev. 2016;2016:1‐18. 10.1155/2016/4350965 PMC4779832 26998193

[29] Davalli P , Mitic T , Caporali A , Lauriola A , D'Arca DR . Cell senescence, and novel molecular mechanisms in aging and age‐related diseases. Oxid Med Cell Longev. 2016;2016:1‐18. 10.1155/2016/3565127 PMC4877482 27247702

[30] Lötvall J , Akdis CA , Bacharier LB , et al. Asthma endotypes: a new approach to classification of disease entities within the asthma syndrome. J Allergy Clin Immunol. 2011;127:355‐360. 21281866 10.1016/j.jaci.2010.11.037

[31] Anderson GP . Endotyping asthma: new insights into key pathogenic mechanisms in a complex, heterogeneous disease. The Lancet. 2008;372:1107‐1119. 10.1016/S0140-6736(08)61452-X 18805339

[32] Yi X , Li Y , Liu H , et al. Inflammatory endotype‐associated airway resistome in chronic obstructive pulmonary disease. Microbiol Spectr. 2022;10(2):e0259321. 35311590 10.1128/spectrum.02593-21 PMC9045194

[33] Ranard BL , Megjhani M , Terilli K , et al. Identification of endotypes of hospitalized COVID‐19 patients. Front Med. 2021;8:770343. 10.3389/fmed.2021.770343 PMC8632028 34859018

[34] Liang LW , Shimada YJ . Endotyping in heart failure—identifying mechanistically meaningful subtypes of disease. Circ J. 2021;85(9):1407‐1415. 34108305 10.1253/circj.CJ-21-0349 PMC9741514

[35] Genkel VV , Shaposhnik II . Conceptualization of heterogeneity of chronic diseases and atherosclerosis as a pathway to precision medicine: endophenotype, endotype, and residual cardiovascular risk. Int J Chronic Dis. 2020;2020:1‐9. 10.1155/2020/5950813 PMC7038435 32099839

[36] Lynch SM , Guo G , Gibson DS , Bjourson AJ , Rai TS . Role of senescence and aging in SARS‐CoV‐2 infection and COVID‐19 disease. Cells. 2021;10:3367. 34943875 10.3390/cells10123367 PMC8699414

[37] Rai TS , Cole JJ , Nelson DM , et al. HIRA Orchestrates a Dynamic Chromatin Landscape in Senescence and is Required for Suppression of Neoplasia. Genes & Development. 2014;28(24):2712‐2725. doi: 10.1101/gad.247528.114 25512559 PMC4265675

[38] Du H , Guo L , Lian J , et al. Establishment of epithelial inflammatory injury model using adult kidney organoids. Life Med. 2024;3(3):lnae022. 39871891 10.1093/lifemedi/lnae022 PMC11749467

[39] Lake BB , Menon R , Winfree S , et al. An atlas of healthy and injured cell states and niches in the human kidney. Nature. 2023;619:585‐594. 37468583 10.1038/s41586-023-05769-3 PMC10356613

[40] Bateman A , Martín M , Orchard S , et al. UniProt: the universal protein knowledgebase in 2023. Nucleic Acids Res. 2023;51:D523‐D531. 36408920 10.1093/nar/gkac1052 PMC9825514

[41] Zabetian A , Coca SG . Plasma and urine biomarkers in chronic kidney disease: closer to clinical application. Curr Opin Nephrol Hypertens. 2021;30:531‐537. 34475336 10.1097/MNH.0000000000000735 PMC8490303

[42] Satake E , Saulnier P , Kobayashi H , et al. Comprehensive search for novel circulating miRNAs and axon guidance pathway proteins associated with risk of ESKD in diabetes. J Am Soc Nephrol. 2021;32:2331‐2351. 34140396 10.1681/ASN.2021010105 PMC8729832

[43] Sayers EW , Bolton EE , Brister JR , et al. Database resources of the national center for biotechnology information. Nucleic Acids Res. 2022;50:D20‐D26. 34850941 10.1093/nar/gkab1112 PMC8728269

[44] Zeng S , Liang Y , Lai S , et al. TNFα/TNFR1 signal induces excessive senescence of decidua stromal cells in recurrent pregnancy loss. J Reprod Immunol. 2023;155:103776. 36495656 10.1016/j.jri.2022.103776

[45] Li Y , Tang M , Han B , et al. Tumor necrosis factor superfamily 14 is critical for the development of renal fibrosis. Aging. 2020;12:25469‐25486. 33231567 10.18632/aging.104151 PMC7803499

[46] Du W , Gao C , You X , et al. Increased proportion of follicular helper T cells is associated with B cell activation and disease severity in IgA nephropathy. Front Immunol. 2022;13:901465. 10.3389/fimmu.2022.901465 PMC9381139 35983053

[47] Sagiv A , Burton DGA , Moshayev Z , et al. NKG2D ligands mediate immunosurveillance of senescent cells. Aging. 2016;8:328‐344. 26878797 10.18632/aging.100897 PMC4789586

[48] van Tuyn J , Jaber‐Hijazi F , MacKenzie D , et al. Oncogene‐expressing senescent melanocytes up‐regulate MHC class II, a candidate melanoma suppressor function. J Invest Dermatol. 2017;137(10):2197‐2207. 28647344 10.1016/j.jid.2017.05.030 PMC5613751

[49] Karin M . How NF‐κB is activated: the role of the IκB kinase (IKK) complex. Oncogene. 1999;18:6867‐6874. 10602462 10.1038/sj.onc.1203219

[50] Sen R , Baltimore D . Multiple nuclear factors interact with the immunoglobulin enhancer sequences. Cell. 1986;46:705‐716. 17114415

[51] Lawrence T . The nuclear factor NF‐κB pathway in inflammation. Cold Spring Harb Perspect Biol. 2009;1:a001651. 20457564 10.1101/cshperspect.a001651 PMC2882124

[52] Zhang X , Wang G , Li M , et al. Both partial inactivation as well as activation of NF‐κB signaling lead to hypertension and chronic kidney disease. Nephrol Dial Transplant. 2024;39(12):1993‐2004. doi:10.1093/ndt/gfae090 38614958

[53] Salminen A , Kauppinen A , Kaarniranta K . Emerging role of NF‐κB signaling in the induction of senescence‐associated secretory phenotype (SASP). Cell Signal. 2012;24:835‐845. 22182507 10.1016/j.cellsig.2011.12.006

